# Supplementary material for: Spatial ecology of little egret (Egretta garzetta) in Hong Kong uncovers preference for commercial fishponds
Source: PeerJ. 2020 Sep 8;8:e9893. doi: 10.7717/peerj.9893 (PMC7485483; doi:10.7717/peerj.9893)
Supplement: Supplemental Information 1 [file peerj-08-9893-s001.docx]

Table S2. Average (±SD) daily home range (50% and 95%), daily travel distance and daily occurrence frequency on fishponds of the tracked little egrets across seasons.

|  | Spring | Summer | Autumn | Winter | Combined |
| --- | --- | --- | --- | --- | --- |
| 95% home range (km^2^) | 8.59±3.67 | 8.49±6.59 | 5.61±10.6 | 11.4±8.97 | 9.26±7.99 |
| 50% home range (km^2^) | 1.83±0.72 | 2.23±1.77 | 1.29±2.35 | 2.59±2.04 | 2.11±1.75 |
| Daily travel distance (km) | 6.76±1.84 | 7.09±3.89 | 4.29±2.51 | 7.21±3.21 | 6.44±2.68 |
| Frequency of occurrence on fishponds | 0.37±0.26 | 0.08±0.10 | 0.36±0.45 | 0.54±0.21 | 0.43±0.27 |
