# Supplementary material for: Spatial ecology of little egret (Egretta garzetta) in Hong Kong uncovers preference for commercial fishponds
Source: PeerJ. 2020 Sep 8;8:e9893. doi: 10.7717/peerj.9893 (PMC7485483; doi:10.7717/peerj.9893)
Supplement: Supplemental Information 2 [file peerj-08-9893-s002.docx]

Table S3. Habitat proportion (%) within the home ranges of tracked Little Egrets.

| Habitats | CHI  01 | HUN01 | HUN02 | HUN03 | HUN04 | PIC  05 | PIC  06 | PIC  07 | PIC  09 | All |
| --- | --- | --- | --- | --- | --- | --- | --- | --- | --- | --- |
| *95% BBMM home range* | | | | | | | | | | |
| Channel | 0 | 2.3 | 9.1 | 4.3 | 0 | 7.5 | 7.0 | 4.4 | 14.9 | 5.5 |
| Fishpond | 87.6 | 46.8 | 50.9 | 21.2 | 54.8 | 43.9 | 36.4 | 21.9 | 58.0 | 42.0 |
| *Gei wai* | 0 | 20.7 | 0.2 | 16.0 | 34.5 | 7.5 | 10.1 | 13.2 | 0 | 7.6 |
| Mangrove | 11.3 | 14.0 | 19.9 | 20.3 | 5.3 | 2.8 | 19.9 | 23.5 | 7.3 | 15.4 |
| Intertidal mudflat | 0 | 11.9 | 9.7 | 31.0 | 0 | 2.5 | 14.8 | 32.4 | 0 | 16.6 |
| Others | 1.1 | 4.3 | 10.2 | 7.2 | 5.4 | 35.7 | 11.7 | 4.5 | 19.9 | 12.9 |
| *50% BBMM home range* | | | | | | | | | | |
| Channel | 0 | 0.0 | 4.4 | 0.5 | 0 | 7.3 | 2.7 | 4.0 | 48.8 | 7.9 |
| Fishpond | 97.7 | 55.4 | 72.6 | 44.5 | 60.8 | 87.8 | 51.9 | 0.2 | 18.0 | 42.9 |
| *Gei wai* | 0.0 | 38.2 | 0.0 | 35.4 | 29.3 | 0.7 | 3.2 | 0.1 | 0 | 7.3 |
| Mangrove | 0.6 | 0.3 | 15.3 | 8.6 | 0.9 | 0.4 | 17.2 | 18.5 | 6.4 | 9.5 |
| Intertidal mudflat | 0 | 0 | 6.5 | 8.7 | 0 | 0 | 21.5 | 75.8 | 0 | 24.5 |
| Others | 1.7 | 6.0 | 1.2 | 2.4 | 9.0 | 3.8 | 3.6 | 1.4 | 26.8 | 7.9 |
